# Effect of speaking valves on early mobilization of patients in intensive care units following tracheostomy: a prospective randomized controlled trial

**DOI:** 10.1016/j.bjorl.2026.101853

**Published:** 2026-07-22

**Authors:** Yujun Chen, Yi Lou, Jiabin Cheng, Na Zhao, Saize Huang, YaJie Zhu, Qinhong Xu

**Affiliations:** a The First Affiliated Hospital of Ningbo University, Intensive Care Unit, Zhejiang Province, China; b The First Affiliated Hospital of Ningbo University, Nursing Department, Zhejiang Province, China

**Keywords:** Tracheostomy, Intensive care unit, Speaking valve, Early mobilisation

## Abstract

**Objectives:**

To evaluate the effect of Speaking Valves (SVs) on early mobilization in Intensive Care Unit (ICU) patients following tracheostomy.

**Methods:**

The participants of this study were patients undergoing tracheotomy who were admitted to ICU of The First Affiliated Hospital of Ningbo University from December 2020 to June 2023. Patients were divided into a test group (n = 47) and a control group (n = 43) based on the random number table method. Further comparison was made on the five-level EuroQol five-Dimensional Questionnaire (EQ-5D-5L) score, swallowing function grading, mobility score, and adverse reactions.

**Results:**

The experimental group demonstrated significantly greater improvement in the primary outcome of mobility assessed by Chelsea Critical Care Physical Assessment Tool (CPAx) (median [IQR] at day-21: 28.0 [25.0‒30.0] vs. control: 22.0 [20.0‒25.0], p < 0.001). The experimental group showed greater improvements in secondary outcomes: EuroQol Visual Analogue Scale (EQ-VAS) (control: 72.40 ± 17.23 to 79.70 ± 11.40; experimental: 73.10 ± 15.23 to 92.34 ± 11.78; p < 0.001), EuroQol five-Dimensional (EQ-5D) scores (control: 0.80 ± 0.21 to 0.90 ± 0.14; experimental: 0.81 ± 0.14 to 0.94 ± 0.13; p < 0.001), and swallowing function grading (control: median 5.00 to 4.00; experimental: 5.00 to 2.00; p < 0.001) following rehabilitation.

**Conclusion:**

The addition of SVs to standard rehabilitation significantly enhanced early mobilization, respiratory function and coughing capacity in patients with tracheostomy. These findings support incorporating SVs into ICU care protocols to prioritize early mobilization, potentially shortening hospital stays and improving functional outcomes.

**Level of evidence:**

Level 3.

## Highlights

• The use of speaking valves in ICU patient’s post-tracheotomy improved respiratory.
• Patients using SVs showed higher EQ-5D-5L scores.
• The findings emphasize the potential of SVs to enhance early mobilization.

## Introduction

Patients in Intensive Care Units (ICUs) generally suffer from severe respiratory dysfunction. Artificial airway management is an essential therapy for stabilizing patients who are critically ill in the ICU.[1,2] Tracheostomy is one of the most common surgical procedures performed in this population. Tracheostomy, a common intervention for patients who are critically ill with respiratory obstruction, involves incising the anterior wall of the cervical trachea and inserting a suitably sized trocar through the incision, allowing the patient to breathe directly through it.[3] Following tracheostomy, due to decreased airway resistance, which prevents the formation of subglottic pressure, a weakened effective cough reflex, closed vocal cords and a disrupted respiratory-swallowing cycle, patients may experience a series of functional impairments. These include difficulties with speech, swallowing, breathing and smell, resulting in a substantial decline in quality of life.[4]

Early mobilization refers to the initiation of physical activity and functional exercises for ICU patients as soon as it is clinically safe to do so.[5] This approach aims to minimize the negative effects of prolonged immobility, such as muscle weakness, joint stiffness and pulmonary complications. Early mobilization encompasses a range of activities, from simple bed mobility to more complex transfers and ambulation, depending on the patient’s condition and stage of recovery. Early mobilization is directly associated with shorter ICU stays and reduced mortality rates.[6]

Multiple rehabilitation strategies are available to reduce postoperative complications in patients who have undergone tracheostomy, such as the use of Speaking Valves (SVs), humidification of the airway, sterile suctioning and swallowing and speech training,[3,4,7] among which the use of SVs is a relatively simple and effective intervention. An SV, also known as a ‘speech valve’, is a one-way ventilation valve that opens during inhalation and closes during exhalation.[4] Various SVs are used in clinical practice and research, including the Passy-Muir valve (flap-type valve), Shiley speech valve, Montgomery valve, Kistner valve, Olympic valve (spring disc valve), Shikani valve (rolling ball valve) and one-way ball valve.[[8], [9], [10], [11], [12], [13]]

There are limited studies in China on the use of SVs in patients with cervical spinal cord injury who have undergone tracheostomy, and debate remains regarding their efficacy in reducing the duration of mechanical ventilation and facilitating earlier extubation.[14] This controversy stems from inconsistent findings in the literature: some studies report that SVs reduce the duration of mechanical ventilation and support earlier extubation,[15] whereas others have found minimal or no benefit.[14,16] These conflicting results underscore the need for further research to clarify the role of SVs in this patient population.

Most existing studies on SVs focus on speech function, swallowing safety and pulmonary ventilation,[17] but there is insufficient investigation into their potential role in promoting early activity by enhancing respiration-motor coupling. Accordingly, this Randomized Controlled Trial (RCT) examines the clinical effect of SVs on early mobilization in patients undergoing tracheostomy, with the aim of informing appropriate intervention strategies and promoting overall recovery.

## Methods

### Participants

**Fig. 1:**
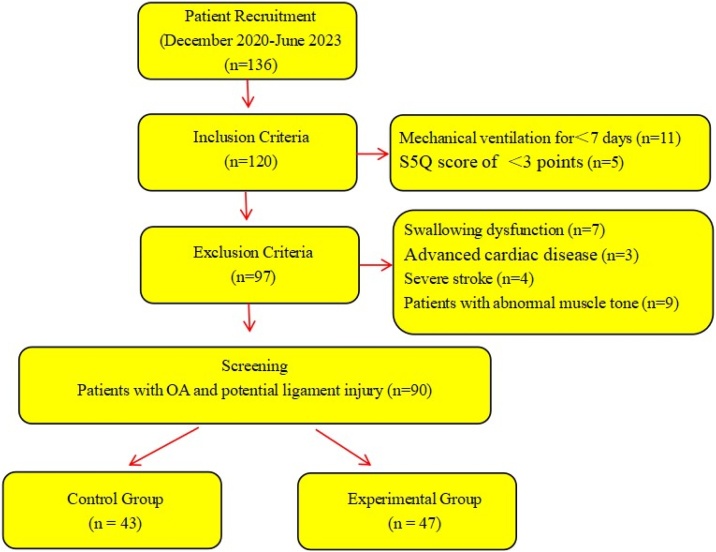
Flow chart of patient recruitment and grouping.

The present study was conducted as a prospective RCT. Patients undergoing tracheostomy who were admitted to the ICU of The First Affiliated Hospital of Ningbo University between December 2020 and June 2023 were included (Fig. 1).

The inclusion criteria were as follows: 1) Patients who had undergone mechanical ventilation for ≥ 7-days; 2) Patients with clear consciousness and a Standardized Five-Question (S5Q) score of > 3-point;[14] and 3) Patients with the ability to perform basic movements or the potential for mobility, as assessed by a physical therapist ‒ an essential criterion for early mobilization. The exclusion criteria were as follows: 1) Patients with swallowing dysfunction prior to tracheotomy; 2) Patients with severe heart, brain or kidney dysfunction, dementia, mental illness or other primary conditions that would substantially impair mobility or the ability to comply with rehabilitation. The dropout criteria were as follows: 1) Patients with poor compliance or voluntary withdrawal and 2) Patients whose condition deteriorated to the extent that they could no longer tolerate participation in the study. Poor compliance was defined as failure to attend scheduled rehabilitation sessions (e.g., missing more than two consecutive sessions without a valid reason) or inability to follow basic rehabilitation instructions due to cognitive or physical limitations not previously identified.

Patients were randomly assigned to an experimental group (n = 47) or a control group (n = 43) using the computer-generated random number table. This study was approved by the Human Ethics Committee of The First Affiliated Hospital of Ningbo University (Approval nº 2020-R245), and all patients or their families provided informed consent.

### Methods

#### Conventional rehabilitation exercises

Patients in both groups received conventional rehabilitation treatment as described in previous research.[18,19] The specific strategies included the following: 1) Ice stimulation training: Ice stimulation was applied to the soft palate and surrounding areas, including the faucial pillars, teeth, cheeks and tongue root. This method was used to stimulate swallowing reflexes during the rehabilitation process. Duration: 5‒10 minutes per session, twice daily. 2) Swallowing exercises: 5‒10 minutes per session, twice daily. 3) Neck extension: Slowly perform forward flexion, backward tilt and lateral extension of the neck to relax the throat muscles and enhance swallowing coordination. 4) Oral exercises: Lip movements ‒ such as pursing the lips, grinning and breathing to strengthen lip muscles. Tongue movements ‒ including touching the upper palate with the tip of the tongue, swinging it left and right and rotating it around the teeth to improve flexibility. Cheek exercises ‒ actions such as puffing out the cheeks and sucking to strengthen buccal muscle control. Voice training: Repetitive pronunciation exercises (such as plosive sounds such as ‘ba’, ‘pa’, and ‘ma’) to promote coordinated throat muscle movement. 5) Respiratory function training: Each session consisted of three sets of 10 repetitions, performed twice daily. Exercises included the use of a breathing trainer, abdominal breathing, pursed lip breathing and playing the flute. All rehabilitation exercises were performed by two experienced rehabilitation physicians.

#### SVs in experimental group

**Fig. 2:**
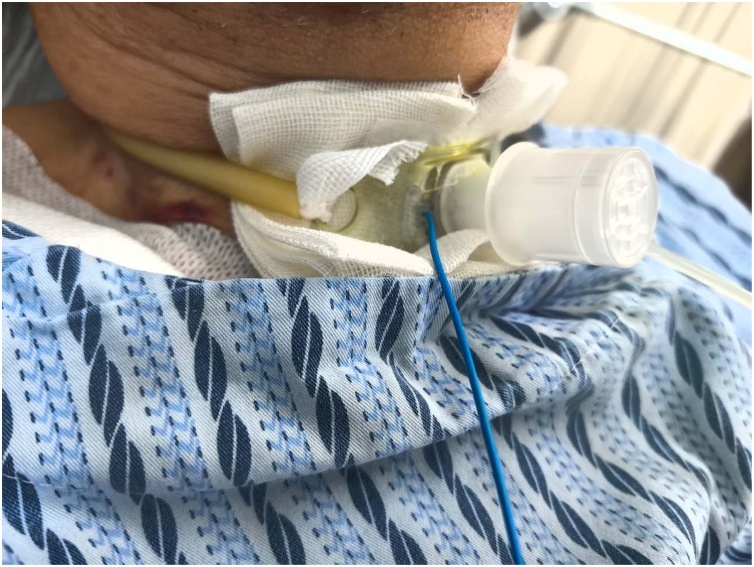
Patients' wearing of speaking valves.

Patients in the experimental group were provided with the use of SVs (RSV20, Run Wide) in addition to the relevant training strategies. Fig. 2 shows patients wearing SVs. Specifically, patients were assisted in positioning themselves (usually in a semi-recumbent position with the bed head raised ≥ 45 °). Following thorough aspiration of the airway and subglottic secretions, oxygen was administered through a nasal catheter at a flow rate of 3‒5 Litres/min. The gas inside the SV was released under tolerable conditions, provided no abnormal changes in vital signs or obvious cough response occurred. After deflation, another suction was performed to keep the trachea unobstructed.

The SV was placed at the entrance of the tracheostomy tube and gently rotated clockwise by the respiratory therapist to prevent sudden dislodgement during coughing or other movements. Following installation, patients were encouraged to speak up and say simple sentences. The try-in was considered successful if the patient could tolerate the device, with relatively stable facial expression, breathing, pulse and blood pressure and oxygen saturation remaining above 95%.

Following a successful try-in, a comprehensive swallowing rehabilitation exercise was initiated while wearing the SV. The first wearing session lasted less than 30-minutes, with the duration gradually increasing based on patient tolerance. Ultimately, patients continued wearing the SV, except during sleep and nebulization treatment. Once the conditions for extubating were met, the tracheostomy tube was removed directly without the requirement for traditional plugging.

### Outcome measures

The primary outcome was Chelsea Critical Care Physical Assessment Tool (CPAx). The secondary outcome, including five-level EuroQol, five-Dimensional Questionnaire (EQ-5D-5 L) scores and swallowing function grading,[20] were recorded before and after rehabilitation. The outcome measures were performed by two blinded researchers who have been trained.

The CPAx was used to evaluate early mobilization, including respiratory function, coughing, bed mobility, transferring from supine to bedside sitting, dynamic sitting, standing balance, transferring from sitting to standing, transferring from bed to chair, walking and grip strength.[21] Each domain was scored from 0 (complete dependence) to 5 (complete independence), with a total score of 50-points. Assessments were conducted at baseline (T0) and on days-7 (T1), 14 (T2) and 21 (T3) after rehabilitation.

The EQ-5D-5 L scale includes five dimensions: mobility, self-care, usual activities, pain/discomfort and anxiety/depression, each rated on a five-level scale. The EuroQol Visual Analogue Scale (EQ-VAS) records patients’ self-rated health on a scale from 0 to 100, where 0 represents the worst possible health and 100 the best. Higher scores indicate a better quality of life. The scale demonstrates good reliability (Cronbach’s α = 0.857).[22]

Swallowing function was assessed using Rosenbek’s penetration-aspiration scale[23] via fibreoptic endoscopic evaluation of swallowing. The patient was seated with the head tilted back, and a scope was inserted through the nasal cavity to observe aspiration during swallowing. A 5 mL volume of low-viscosity food colourant was used to trigger the swallowing reflex. Low-viscosity colourant was selected as it closely mimics saliva and is less likely to adhere to the airway compared with thicker substances, enhancing safety during the assessment.[24] Higher scores on the 1‒8 scale indicates poorer swallowing safety.

The Fugl-Meyer scale[25] was used to assess the motor function of the upper, with maximum scores of 66 and 34, respectively. Higher scores reflect stronger motor function.

Adverse events such as hypotension, tachycardia, desaturation or arrhythmias were recorded to evaluate the safety of the mobilization protocol.

### Statistical analysis

The statistical analysis of this study was completed using SPSS 26.0 software (IBM, Armonk, NY, USA). Measurement data in the general data analysis were expressed as mean ± standard deviation (x̄ ± s). An independent sample *t*-test was used for grouped data, a paired *t*-test for paired data and the Chi-Square (χ^2^) test for counting data. For CPAx score, Generalized Estimating Equations (GEE) were employed to account for repeated measures and to evaluate group, time, and interaction effects. The between-group comparisons of CPAx scores at each time point were performed using Mann-Whitney *U*-tests. The p < 0.05 indicated a statistically significant difference.

## Results

### General data

As shown in Table 1, there was no statistically significant difference in age, gender, S5Q score, CPAx score or medical diagnosis between the groups (all p > 0.05), suggesting inter-group comparability.

**Table 1:** Comparison of general data between groups (x ± s).

Items	Control group (n = 43)	Experimental group (n = 47)	*t*/χ^2^/*Z*	p
Age/years	58.79 ± 15.268	61.38 ± 14.477	−0.825	0.412
Gender/cases			0.440	0.230
Males	28 (65.12)	36 (76.60)		
Females	15 (34.88)	11 (23.40)		
BMI	23.48 ± 2.11	23.89 ± 2.01	0.511	0.611
Smoking history	19	17	0.601	0.438
Drinking history	24	20	1.580	0.209
hypertension	9	7	0.560	0.454
Diabetes	4	6	0.035	0.852
Upper limb motor function	40.28 ± 5.19	40.55 ± 5.42	0.429	0.669
S5Q/points	4.07 ± 0.799	4.04 ± 0.779	0.163	0.871
CPAx score	15.0 (13.0, 18.0)	16.0 (13.0, 19.0)	−0.665	0.508
Medical diagnosis/Cases			‒a	0.833
Cerebral hemorrhage	14 (32.56)	14 (29.79)		
Cerebral infarction	4 (9.30)	4 (8.51)		
Craniocerebral trauma	6 (13.95)	6 (12.77)		
Multiple trauma	4 (9.30)	9 (19.15)		
Cardiac disease	6 (13.95)	4 (8.51)		
Others	9 (20.93)	10 (21.28)		

BMI, Body Mass Index; S5Q, Standardized five-Question; CPAx, Chelsea Critical Care Physical Assessment Tool.

a Compared using Fisher's exact probability test.

### Comparison of early mobilization of CPAx between groups

**Fig. 3:**
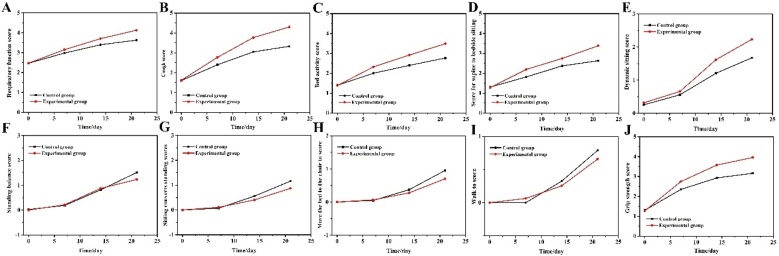
Changes in CPAx scores of the two groups of patients. (A) Respiratory function; (B) Coughing function; (C) Bed mobility; (D) Transferring from supine position to bedside sitting position; (E) Dynamic sitting position; (F) Standing balance; (G) Moving transferring from sitting position to standing position; (H) Transferring from bed to chair; (I) Walking; and (J) Grip strength.

The generalized estimating equation revealed notable inter-group interaction effects over time on respiratory function, coughing function, bed mobility, transferring from supine to bedside sitting, walking and grip strength, justifying the use of simple effects analysis (p < 0.05). Time-based analysis demonstrated substantial improvements in all indicators for both groups (p < 0.001), whereas between-group analysis revealed statistically significant differences in respiratory function, coughing function, bed mobility, transferring from supine to sitting and grip strength (p < 0.05, Table 2 and Fig. 3).

**Table 2:** The generalized estimating equation for CPAx score.

Indicators	Between-group		Time		Interaction	
	Wald	p	Wald	p	Wald	p
Respiratory function	3.847	0.050	428.379	<0.001	15.402	0.002
Coughing function	9.535	0.002	646.273	<0.001	31.764	<0.001
Bed mobility	5.402	0.020	277.782	<0.001	12.276	0.006
Transferring from supine position to bedside sitting position	5.860	0.015	240.659	<0.001	17.788	<0.001
Dynamic sitting position	2.867	0.090	268.992	<0.001	7.647	0.054
Standing balance	0.131	0.717	102.947	<0.001	3.053	0.384
Moving transferring from sitting position to standing position	0.644	0.422	48.135	<0.001	2.403	0.493
Transferring from bed to chair	0.527	0.468	40.871	<0.001	1.676	0.642
Walking	0.096	0.757	33.991	<0.001	8.164	0.043
Grip strength	10.517	0.001	865.442	<0.001	27.955	<0.001
Total score	2.695	0.101	287.390	<0.001	7.183	0.066

CPAx, Chelsea Critical Care Physical Assessment Tool.

Total CPAx scores at different time points are presented in Table 2. At baseline (T0), no significant difference existed between groups (15.0 [13.0‒18.0] vs. 16.0 [13.0‒19.0], *Z* = -0.665, p = 0.508). Between-group comparisons revealed significantly higher CPAx scores in the experimental group at all follow-up time points: day-7 (T1: 19.0 [17.0‒22.0] vs. 17.0 [15.0‒19.0], *Z* = -3.245, p = 0.001), day-14 (T2: 24.0 [22.0‒27.0] vs. 20.0 [18.0‒23.0], *Z* = -4.812, p < 0.001), and day-21 (T3: 28.0 [25.0‒30.0] vs. 22.0 [20.0‒25.0], *Z* = -5.927, p < 0.001), indicating accelerated mobility recovery with SV intervention (Table 3).

**Table 3:** CPAx scores at baseline and during follow-up.

Time point	Control group (n = 43)	Experimental group (n = 47)	Z-value	p-value
T0	15.0 (13.0, 18.0)	16.0 (13.0, 19.0)	−0.665	0.508
T1	17.0 (15.0, 19.0)	19.0 (17.0, 22.0)^a^, ^b^	−3.245	0.001
T2	20.0 (18.0, 23.0)^a^	24.0 (22.0, 27.0)^a^, ^b^	−4.812	<0.001
T3	22.0 (20.0, 25.0)^a^	28.0 (25.0, 30.0)^a^, ^b^	−5.927	<0.001

CPAx, Chelsea Critical Care Physical Assessment Tool.

a Significant within-group improvement vs T0 (p < 0.05, Wilcoxon signed-rank).

b Significant between-group difference (p < 0.05, Mann-Whitney *U*-test).

### EuroQol-5D-5L score and swallowing function grading before and after treatment

As shown in Table 3, there was no statistically significant difference in EQ-VAS score, EuroQol five Dimensions (EQ-5D) score or swallowing function grading between the two groups before rehabilitation (all p > 0.05). Both groups demonstrated improvement in EQ-VAS score, EQ-5D score and swallowing function grading following rehabilitation, compared with pre-rehabilitation levels (all p < 0.05).

Moreover, the inter-group comparison at 21-days post-rehabilitation revealed that EQ-VAS (79.70 ± 11.40 vs. 92.34 ± 11.78) and EQ-5D (0.90 ± 0.14 vs. 0.94 ± 0.13) scores in the experimental group were higher than those in the control group, whereas swallowing function grading (4.00 vs. 2.00) was lower than that in the control group (all p < 0.05) (Table 4). Patients in both groups did not experience considerable adverse reactions during rehabilitation, indicating good recovery.

**Table 4:** EQ-5D-5L score and swallowing function grading before and after treatment.

Indicators	Control group (n = 43)	Experimental group (n = 47)	*t*-value	p-value
EQ-VAS score				
Before	72.40 ± 17.23	73.10 ± 15.23	0.789	0.433
After	79.70 ± 11.40	92.34 ± 11.78	3.245	0.001
*t-*value	5.321	14.321		
p-value	<0.001	<0.001		
EQ-5D score				
Before	0.80 ± 0.21	0.81 ± 0.14	0.511	0.611
After	0.90 ± 0.14	0.94 ± 0.13	3.421	0.001
*t* value	3.762	6.424		
p-value	<0.001	<0.001		
Swallowing function grading				
Before	5.00 (3.50, 6.00)	5.00 (2.00, 5.00)	0.250	0.803
After	4.00 (2.00, 5.00)	2.00 (1.00, 4.00)	2.680	0.007
*Z-*value	4.529	12.453		
p-value	<0.001	<0.001		

EQ-VAS, EuroQol Visual Analogue Scale; EQ-5D, EuroQol five-Dimensional.

^a^Statistically significant difference compared to that before rehabilitation.

## Discussion

In this RCT, we investigated the impact of SVs on early mobilization in ICU patients following tracheostomy. The results indicate that both groups exhibited improvements in EQ-VAS score, EQ-5D score and swallowing function grading following rehabilitation. Additionally, SVs substantially improve, enhanced respiratory and coughing functions, and facilitated early mobilization in basic activities such as bed mobility and grip strength. These improvements were observed in the short term, highlighting the potential of SVs to enhance recovery in the early stages of post-tracheostomy.

As noted in a prior systematic review, coughing ability is a key indicator associated with extubating in mechanically ventilated patients. The cough reflex is essential for airway self-cleaning, as ineffective coughing may lead to secretion accumulation and increased airway resistance, resulting in breathing difficulty and subsequent impairment of respiratory function.[26] The sensory receptors of patients undergoing tracheostomy remain in a closed state, and the glottis loses its role in coughing. Speaking valves enable sensory receptors in the pharyngolaryngeal mucosae to regain airflow stimulation from the upper respiratory tract, which can subsequently reshape subglottic pressure, reconstruct the glottic-stop reflex and restore the cough reflex. In a recent study, the ability to tolerate 4 -h SV wearing was identified as an independent predictor of successful tracheostomy tube extubating, with a 100% success rate in 96.7% of patients who could tolerate 4 -h SV use and met additional extubating criteria.[27]

Multiple studies have also demonstrated that wearing SVs can correct pathological breathing, reconstruct the cough reflex, improve respiratory muscle strength, gradually restore respiratory function and substantially increase tidal volume.[[28], [29], [30]] Similarly, the present study provides evidence supporting the effects of SVs on respiratory and coughing functions. The data indicate that the use of SVs can enhance respiratory and coughing performance, facilitate successful extubating of the tracheostomy tube and accelerate recovery.

According to current expert consensus, early activity can effectively promote the recovery of neuromuscular function in ICU patients.[31] However, a survey by Lene Lehmkuhl reported that mechanically ventilated ICU patients had limited mobility, spending 20 -hs per day lying in bed, 3 -hs sitting and only 1 -h engaged in other activities.[32] A prospective study conducted in Brazil in 2020 was the first to assess whether SVs could improve early mobilization in ICU patients, using the Perme Intensive Care Unit Mobility Score across seven aspects. The results showed that patients wearing SVs experienced an overall improvement in mobility, with ‘transfer’ being the most substantially improved factor.[33] However, this study included only 18 patients, lacked a control group and had substantial limitations.

Our results demonstrate that SV intervention significantly improved overall CPAx scores at all follow-up time points (days-7, -14, and -21), with the experimental group showing better gains than controls. This enhancement in early mobilization may be attributed to the beneficial effects of SVs on respiratory and coughing functions, which facilitate increased activity tolerance. Specifically, SV use may increase end-inspiratory and expiratory lung volumes, thereby reducing the work of breathing[34] and enabling patients to engage more effectively in mobilization activities. Consistent with this mechanism, we observed pronounced improvements in foundational mobility components (bed mobility and supine-to-sitting transfers) from day-14 onward. However, complex functions like transfers and gait showed less pronounced gains, likely due to the cumulative effects of ICU-acquired weakness requiring longer rehabilitation periods beyond our 21-day observation window.

Grip strength, a simple and objective index of muscle strength, can be used to assess patients’ strength and mobility. Mechanically ventilated ICU patients in China tend to have relatively low muscle strength.[35] A recent study found that grip strength in ICU patients was associated with mental state and quality of life, with lower grip strength observed in patients experiencing higher levels of anxiety and depression or poorer quality of life.[36]

Patients are prone to negative emotions when basic physiological and psychological needs are unmet. There is a considerable difference in people’s ability to understand patients who are unable to speak, and the use of SVs greatly improved the success rate of doctor-patient and nurse-patient communication.[37] In this study, overall grip strength was low in all patients, but it improved in those wearing SVs by day-7 of intervention. Nonetheless, the connection between communication and exercise efficiency appears less direct. The focus remains on the rehabilitation protocol and SV use, which are more relevant to the observed improvements in grip strength and limb mobility.

It is important to acknowledge the limitations of this study. First, although patients tolerated the SV well and all participants completed rehabilitation as planned without dropouts, the use of SVs required additional time and nursing labour. Therefore, clinical recommendations for SV use should consider the extra resources needed for their management. Second, the single-center design and short follow-up period limit the generalizability of the findings. These factors suggest the long-term effects of SVs on recovery remain to be clarified.

## Conclusion

In conclusion, this study suggests that the use of SVs alongside standard rehabilitation substantially enhances early mobilization, respiratory function and coughing capacity in patients with tracheostomy. Speaking valves appear to be a safe and effective adjunct to tracheostomy care and are recommended for clinical use. Future studies with larger sample sizes and extended follow-up periods are warranted to validate these findings.

## ORCID ID

Yujun Chen: 0009-0003-9070-3315

Yi Lou: 0009-0003-8176-4167

Jiabin Cheng: 0009-0004-1697-128X

Na Zhao: 0009-0007-4549-6811

Saize Huang: 0009-0009-0538-5046

YaJie Zhu: 0009-0005-9927-1920

Qinhong Xu: 0009-0006-5136-245X

## Funding

This work was supported by Study of speech valve combined with comprehensive swallowing rehabilitation for the prevention of aspiration pneumonia in critically ill patients with tracheotomy (grant nºH2020YJ008).

## Ethics approval and consent to participate

The study adhered to the principles outlined in the Declaration of Helsinki and obtained ethical approval from the Clinical Research Ethics Committee of the First Affiliated Hospital of Ningbo University (Approval nº2020-R245).

## Conflicts of interest

The authors declare no conflicts of interest.

## Data availability statement

All data generated or analyzed during this study are included in this article.
